# Modification of Chitosan with (−)-Gossypol and (−)-Gossypol Acetic Acid Using Free-Radical Grafting Method

**DOI:** 10.3390/ijms262311721

**Published:** 2025-12-03

**Authors:** Anna Hlukhaniuk, Małgorzata Świętek, Anna Kołodziej, Aleksandra Wesełucha-Birczyńska, Andrii Mahun, Libor Kobera, Daniel Horák

**Affiliations:** 1 Department of Polymer Particles, Institute of Macromolecular Chemistry, Czech Academy of Sciences, Heyrovského nám. 2, 162 00 Prague, Czech Republic; hlukhaniuk@imc.cas.cz (A.H.); mahun@chalmers.se (A.M.); kobera@imc.cas.cz (L.K.); 2 Faculty of Science, Charles University, Albertov 2038, 128 00 Prague, Czech Republic; 3 CEISAM Institute UMR 6230, CNRS, Nantes University, F-44000 Nantes, France; 4 Faculty of Chemistry, Jagiellonian University, Gronostajowa 2, 30-387 Krakow, Poland; anka.kolodziej@uj.edu.pl (A.K.); birczyns@chemia.uj.edu.pl (A.W.-B.)

**Keywords:** chitosan derivatives, gossypol, polyphenols, free-radical grafting

## Abstract

One of the approaches to increase bioavailability and stability of hydrophobic biologically active compounds is their incorporation into polymer backbone. This work deals with the modification of chitosan (CS) with gossypol (GS), a phenolic compound with confirmed anticancer properties, by the free-radical grafting method. The series of the CS derivatives with increasing content of GS were prepared using pure GS or gossypol acetate (GSA) and compared to the control CS (cCS). The starting CS, cCS, and GS-containing derivatives were characterized using Fourier-transform infrared (FTIR), Raman, and ^13^C ssNMR spectroscopies; elemental and thermogravimetric analysis to evaluate the influence of the radicals and GS on the properties of polymers was performed. The Folin–Ciocalteu (F-C) and 2,2-diphenyl-1-picrylhydrazyl (DPPH) methods were used to evaluate antioxidant properties of GS-modified CSs. Additionally, the polymer solubility and the specific viscosity of the solutions were determined. The content of GS in polymers raised proportionally with increasing amount of GS added to the reaction mixture, thereby enhancing the ability to scavenge free radicals. The type of GS used (GS or GSA) in polymers affected the degree of CS crosslinking (higher for pure GS), polymer solubility (lower for pure GS), the amount of grafted GS (~20% higher for GSA), and antioxidant properties in favor of GSA.

## 1. Introduction

The nature-derived phenolic compounds are known for their antioxidant, anti-inflammatory, antibacterial, antiviral, and anticancer properties [1]. As a result, they currently attract considerable attention as substances with high potential for various biomedical applications. Among them, gossypol (*Gossypium* sp.; GS), a polyphenolic compound found in cotton, especially in cottonseeds, has been shown to be highly effective against breast, pancreatic, colon, cervical, lung, and prostate cancer cells [2]. The anticancer activity of GS is complex and multidirectional. GS disrupts several cellular mechanisms, inducing cell apoptosis and autophagy, inhibiting cell viability, and modulating their signaling pathways (including Nrf2/ARE, NF-κB, FAK). The activation of cellular apoptosis in cancer cells is the result of the suppression of antiapoptotic Bcl-2 proteins, the activation of caspase-dependent cell death pathways, interference with the DNA replication process, disruption of telomerase activity, and stimulation of reactive oxygen species (ROS) generation. Abnormally high concentrations of ROS induce oxidative stress in cells, causing a shortage of adenosine triphosphate, and consequently mitochondrial dysfunction and damage to cellular structures, leading to cell death. Moreover, GS also modulates immune systems of cancer cells, facilitating their recognition by T- and natural killer cells. However, it has to be noted that GS, being a defense molecule in cotton, also shows intrinsic toxicity as a contamination in the human food chain, especially when accumulated over a long period of time [2,3]. Free GS is associated with gastrointestinal adverse effects, anorexia, respiratory diseases, hemorrhagic inflammation, etc. Moreover, due to the GS ability to impair male fertility, it has been proposed as a contraceptive drug. Binding GS to a polymer significantly decreases its toxicity.

**Figure 1:**
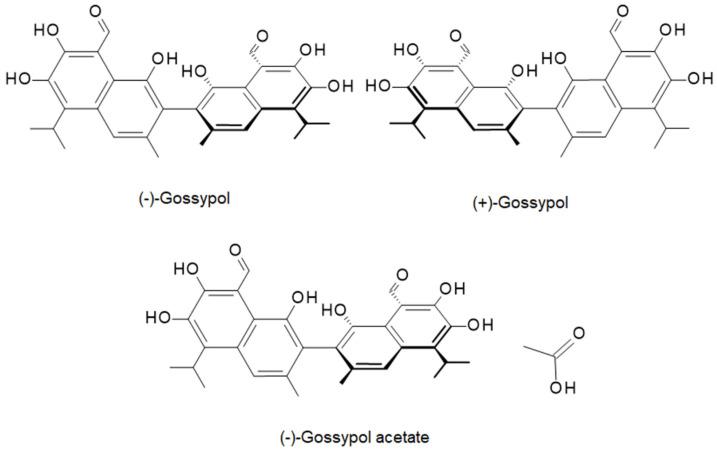
The chemical structure of GS enantiomers and GS acetate (GSA).

From the chemical point of view, GS is composed of two naphthalene aromatic rings joined by a single bond (Figure 1) [3]. Hydroxyl and aldehyde functional groups attached to the aromatic rings of GS are decisive for its biological function; however, aldehyde groups, along with alkyl groups, also contribute to the hydrophobicity of GS. Importantly, GS exists in two enantiomeric forms, (+) and (−), of which the (−) form exhibits stronger anticancer properties [2]. Besides pure GS, its acetate salt (GSA) is also widely investigated due to its enhanced solubility, which translates into better bioavailability. Even so, compared to water-soluble phenolic compounds, the solubility of GSA remains low and is a major obstacle hindering its use in biomedical applications. In addition, covalent attachment of GS to a polymeric carrier offers a promising strategy to minimize GS systemic toxicity.

One of the most commonly used ways to increase the bioaccessibility of hydrophobic, biologically active molecules is their chemical incorporation into the backbone of a hydrophilic polymer [4]. Besides improved bioavailability, this contributes to the enhanced stability of molecules, especially those which are light- and oxygen-sensitive. Examples of hydrophilic polymers are polysaccharides, which are formed by monosaccharide units linked by glycosidic bonds. Polysaccharides that are commonly found in plants and animals are characterized by great diversity in terms of composition, functional groups, and molecular weight (*M*_w_), which makes them adaptable for a wide range of biomedical applications [5]. These include vaccine modulators, hemostatic and immunomodulatory agents, demulcents, plasma substitutes, matrices for tissue engineering scaffolds, and drug carriers. Chitosan (CS) is an example of a polysaccharide widely used in medicine [6]. This animal-derived aminopolymer is obtained by deacetylation of chitin. CS is biodegradable, biocompatible, and characterized by antimicrobial, antifungal, and antitumor activities, and the ability to activate macrophages. Antitumor activity of CS is based on activating dendritic cells, triggering natural killer cells via regulation of IFN-γ production (particularly in leukemia cells) [7], inducing apoptosis [8], and by promoting oxidative stress in response to increased ROS levels [9]. Furthermore, CS and its derivatives have been extensively tested for drug delivery systems. For example, amphoteric films of CS derivatives containing 5-fluorouracil [10], self-assembled cationic nanoparticles from amphiphilic chitosan derivatives as a vector for codelivery of doxorubicin and genes [11], micelles based on *N*-(2,3-dihydroxypropyl)-CS-cholic acid for paclitaxel delivery [12], and disulfide-crosslinked CS-stearic acid nanoparticles for dual drug delivery [13] have been developed. The great advantage of CS over other polysaccharides is the presence of amino and primary and secondary hydroxyl functional groups in its structure. Especially, amino groups are often chosen as a site of chemical modification of CS, aiming not only to increase its water solubility, but also biological functionality [14]. When it comes to the modification of CS with phenolic compounds, several strategies have already been proposed, e.g., laccase- or tyrosinase-mediated enzymatic grafting, carbodiimide-activated conjugation via esterification or amidation, and free-radical grafting [15]. Among the above-mentioned approaches, the free-radical grafting method is associated not only with the incorporation of a phenolic compound into the CS backbone, but also with its degradation, leading to enhanced solubility. Another indisputable advantage of the free-radical grafting often using a hydrogen peroxide-ascorbic acid mixture is its cost-effectiveness, mild reaction conditions at room temperature (so-called green synthesis), and the ability to graft a wide range of molecules. This is particularly beneficial in the case of phenolic compounds, whose aromatic ring hydroxyl groups can stabilize free radicals through resonance. On the other hand, the amino and hydroxyl groups of CS are suitable for binding GS via free-radical reaction.

The aim of this work was to modify CS polysaccharide with various amounts of two forms of GS, pure GS and GSA, using free-radical grafting, which has not yet been reported. We evaluated whether the form of GS affects the grafting efficacy and the physicochemical and antioxidant properties of the final derivatives.

## 2. Results and Discussion

**Figure 2:**
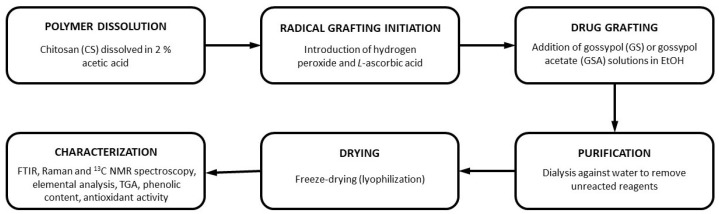
Schematic representation of the preparation of GS-modified CS derivatives via free-radical grafting.

**Figure 3:**
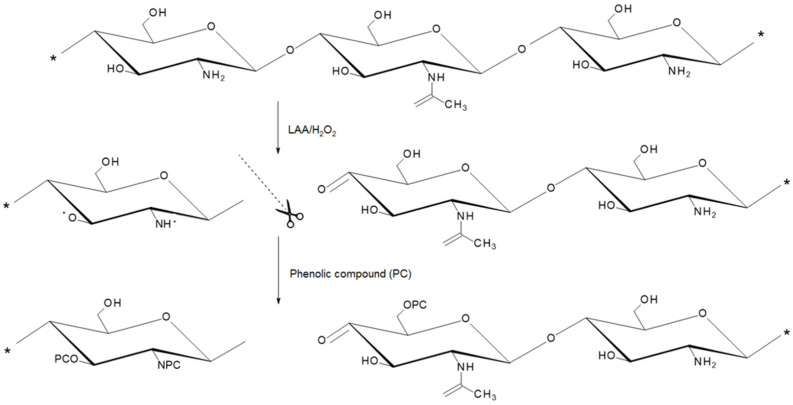
Mechanism of free-radical grafting of phenolic compounds (PC) on CS. The scissors indicate the site of hydroxyl radical-induced depolymerization of CS [20]; * represents the binding site of additional monomeric units.

To prepare GS-modified CSs, a simple green free-radical grafting approach was chosen as it does not require harsh reaction conditions and does not produce toxic byproducts. Moreover, it has already been reported as a convenient way to covalently incorporate various phenolic compounds into the CS backbone [16,17,18]. The overall modification process is shown in Figure 2, which highlights the main stages of radical initiation, modification with GS or GSA, and subsequent purification. In this method, a grafting process is divided into two stages (Figure 3). In the first step, a polymer is treated with an *L*-ascorbic acid (LAA)/H_2_O_2_ redox pair to produce polymer macroradicals. Then, the macroradicals react with a phenolic compound in the second step.

### 2.1. Characterization of Highly Viscous Chitosan (hvCS) and Control Chitosan (cCS)

**Figure 4:**
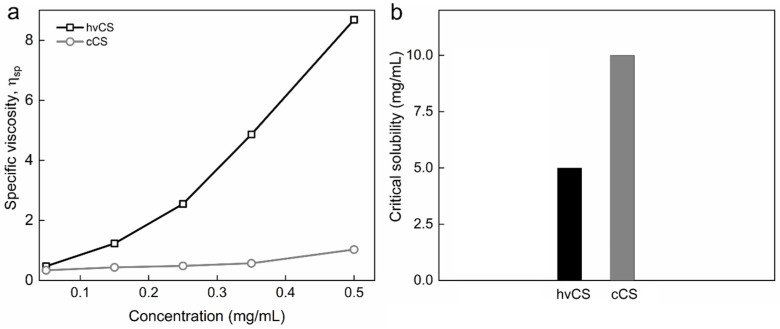
(**a**) Dependence of specific viscosity on the concentration of hvCS and cCS in solution in 2% AA and (**b**) critical solubility of hvCS and cCS at RT.

To evaluate the effect of the LAA/H_2_O_2_ redox pair on the polymer itself, to investigate the properties of the polymer subjected to the insertion of the phenolic compounds, and to evaluate the success of GS or GSA substitution, cCS in the absence of GS was used as a control. After adding LAA/H_2_O_2_ to the hvCS solution, a rapid decrease in its viscosity was observed, which was assigned to the degradation of the polymer. The significant difference in *M*_w_ values between cCS and hvCS was reflected in very distinct specific viscosity (*η*_sp_) values obtained for solutions of these polymers, especially at concentration of 2.5 mg/mL or higher (Figure 4a). Of note, a previous study showed that the *M*_w_ of hvCS was 5.4 × 10^3^ kDa; after free-radical grafting, it decreased by three orders of magnitude [16]. In addition, the critical solubility of cCS was two-fold higher compared to that of hvCS (Figure 4b). Depolymerization of CS by LAA/H_2_O_2_ is known to depend on the concentrations of CS, LAA, and H_2_O_2_, initial pH, temperature, CS contamination with heavy metals. The depolymerization was assigned to the action of hydroxyl and/or ascorbate radicals [19,20,21]. Importantly, the mechanism of ascorbate radical-induced degradation of CS differs from that reported for hydroxyl radicals [20]. The anionic character of the ascorbate radical predisposes it to electrostatic interaction with cationic amino groups, as well as to hydrogen abstraction from the C-4 and C-6 positions. The loss of cationic amino groups later leads to the destruction of α(1 → 4) glycosidic bonds associated with the formation of unsaturated bonds. In contrast, hydroxyl radical abstracts hydrogen at C-4 position of CS, causing breaking of the α(1 → 4) glycosidic bond and formation of C=O. In addition to the scission of α(1 → 4) glycosidic bonds in CS, the deamination and ring-opening oxidation of CS as a result of its treatment with LAA/H_2_O_2_ have also been reported [19,20,21].

**Figure 5:**
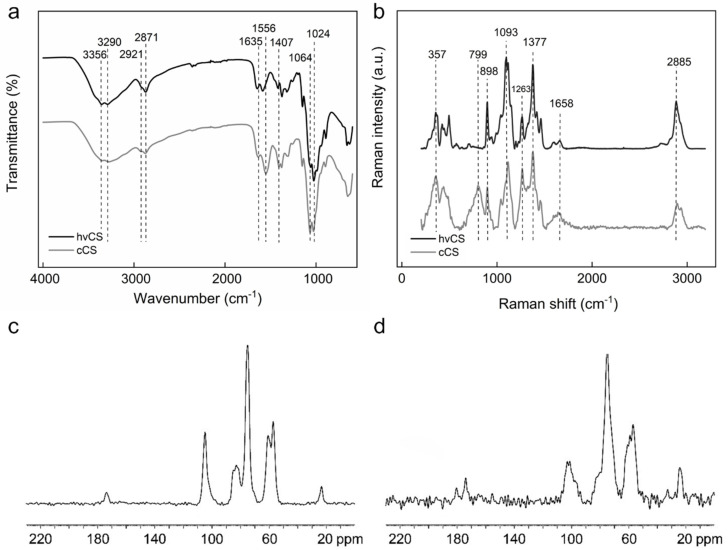
(**a**) ATR-FTIR and (**b**) Raman spectra of hvCS and cCS. ^13^C CP/MAS NMR spectra of (**c**) hvCS and (**d**) cCS.

The ATR-FTIR spectrum of hvCS showed strong bands at 3356 and 3290 cm^−1^, originating from O-H and N-H stretching (Figure 5a) [22]. Peaks at 2921 and 2871 cm^−1^ were attributed to C-H stretching and CH_3_ symmetrical stretch typical for polysaccharides. Bands at 1650 and 1590 cm^−1^ were assigned to the carbonyl stretching of amide I and N-H bending. CH bending and CH_3_ symmetrical deformations were observed at 1419 and 1375 cm^−1^, respectively. Stretching of the acetyl group in amide III was registered at 1317 cm^−1^. Bands at 1149 and 1064 cm^−1^ were attributed to the C-O-C angular deformation and asymmetrical stretching. The band at 1024 cm^−1^ originated from the asymmetric stretch of C-O in a pyranose ring, while the band at 896 cm^−1^ corresponded to the C-H ring stretch. In the ATR-FTIR spectrum of cCS, compared to the spectrum of hvCS, a shift to lower wavenumbers was observed for the bands attributed to the carbonyl stretching of amide I (1650–1635 cm^−1^), N-H (1590–1552 cm^−1^), and C-H bending (1419–1407 cm^−1^; Figure 5a). These changes likely originated from the vibration of the carboxylate ion, confirming the presence of residual acetic acid (AA). Furthermore, the intensity of the N-H band at 1556 cm^−1^ increased, while the intensity of the bands >2800 cm^−1^ decreased, implying structural changes in these groups due to the LAA/H_2_O_2_ treatment [23].

The Raman spectrum of hvCS showed typical CS bands, including ν(CH_2_) at 2885 cm^−1^, ν(C=O) at 1658 cm^−1^, δ(CH_2_) + δ(CH) + δ(OH) + ν(pyranose ring) at 1377 cm^−1^, ν(C–O) + ν(C–C) + δ(OH…O) + ρ(CH_3_) at 1263 cm^−1^, ν(C-O-C) + ν(pyranose ring) + ν(C-OH) + ν(C-CH_2_) + δ(CH) + ρ(CH_2_) + ρ(CH_3_) at 1093, 1114, and 1146 cm^−1^, ν(pyranose ring) + ρ(CH_2_) at 898 cm^−1^, and γ(OH) + γ(pyranose ring) at 357, 424, and 444 cm^−1^ (Figure 5b) [24]. In the spectrum of cCS, the intensity of the 2936 and 1377 cm^−1^ bands increased compared to those of hvCS, indicating a higher proportion of CH_3_ to CH_2_ groups (Figure 5b). This was likely related to the contamination of the cCS with AA.

The ^13^C CP/MAS NMR spectrum of hvCS displayed a set of signals inherent to CS (Figure 5c). Apart from the signals corresponding to *D*-glucosamine (57–105 ppm), there were two distinct signals at 23.5 ppm (methyl carbon) and 173.8 ppm (carbonyl carbon) corresponding to acyl groups of *N*-acetyl-*D*-glucosamine units. Compared to the ^13^C CP/MAS NMR spectrum of hvCS, the spectrum of cCS showed the same peaks; however, the signals were noisier (Figure 5d).

Generally, in the structure of CS, three types of functionalities can be distinguished: amino, acetyl, and hydroxyl (primary and secondary) groups. Among those, amino groups are the most important when it comes to CS solubility and biological properties, as well as strategies of its modification [25]. Due to the nucleophilicity of amino groups, they are prone to react with, e.g., aldehydes, carboxylic acids, and their derivatives [26]. For this reason, the degree of deacetylation (*DDA*), defining the ratio of amino groups to acetylated ones, is an important parameter. *DDA* of hvCS calculated from the ^13^C CP/MAS NMR spectra was 83 ± 2%. Elemental analysis, providing information on the content of C, N, and H in polymers, was used as a complementary method to determine *DDA* (Table 1). *DDA* calculated from the C/N ratio was 80%, which agreed with spectroscopic data. In turn, the C/N ratio in cCS was slightly lower, *DDA* = 75%.

**Figure 6:**
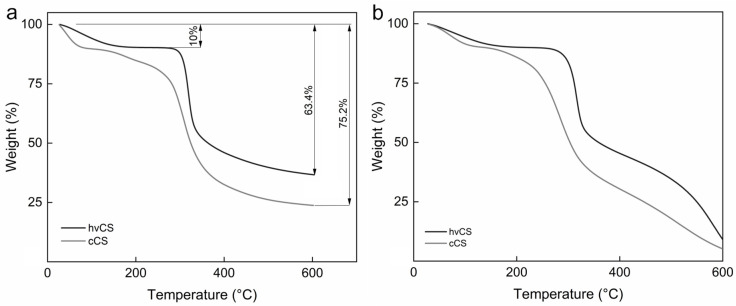
TGA curves of hvCS and cCS in (**a**) nitrogen and (**b**) air atmosphere.

Thermal decomposition of hvCS recorded in ambient atmosphere proceeded in two stages. The first stage, observed up to 150 °C, was attributed to the elimination of the adsorbed and bound water, resulting in a mass loss of 10% (Figure 6a) [27]. The second decomposition stage initiated at 300 °C was assigned to the thermal degradation of the polymer, including its dehydration, deamination, deacetylation, depolymerization, and opening of pyranose units. This stage was associated with the mass loss of ~40%, and was followed by further slow mass reduction up to 600 °C. This was assigned to the degradation of oxygen-containing functional groups and the structural arrangement of carbons [28]. In an oxidative atmosphere up to ~400 °C, the thermal decomposition of hvCS proceeded similarly to that in nitrogen with the burning of the polymer degradation products contributing to a huge mass loss (Figure 6b). In contrast, thermal degradation of cCS under both tested conditions proceeded in three stages, with an additional step after water removal attributed to the degradation of the products of CS depolymerization, as well as distinct crystallinity and *M*_w_ of cCS compared to those of the untreated polymer (Figure 6a,b) [29,30]. Moreover, the onset of polymer degradation was significantly shifted to lower temperatures.

### 2.2. Characterization of the GS-Modified CS Derivatives

**Figure 7:**
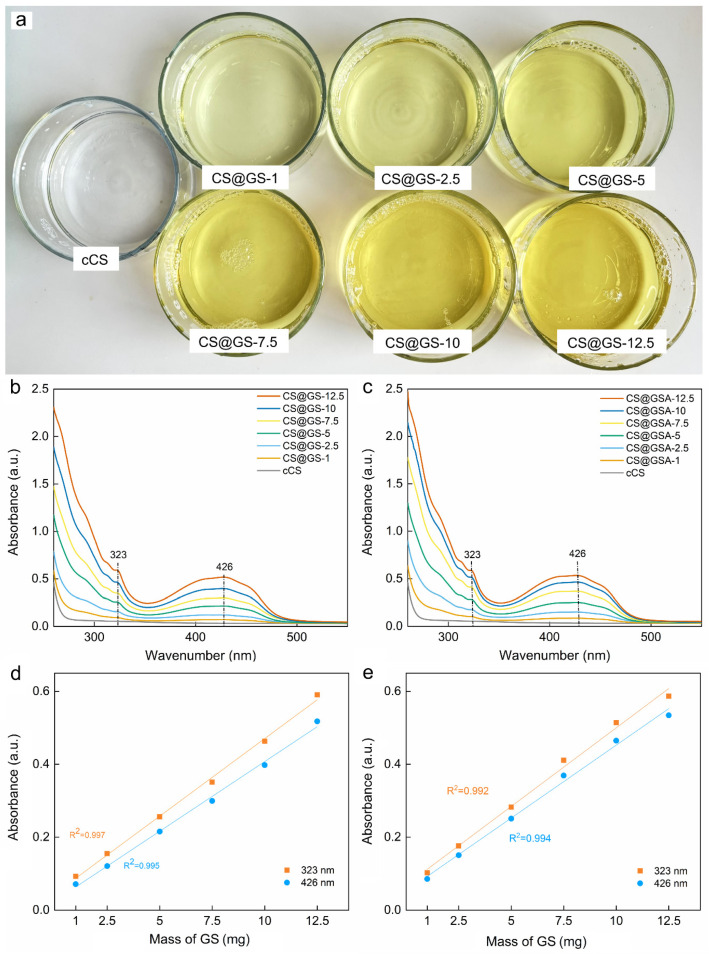
(**a**) Solutions of cCS and CS@GS derivatives after dialysis against water. UV–Vis spectra of (**b**) CS@GSs and (**c**) CS@GSAs in 2% AA at RT. Dependence of absorbance on mass of GS used for preparation of (**d**) CS@GS and (**e**) CS@GSA derivatives.

In contrast to CS, which appears as a white or light-yellow powder producing transparent solutions, GS is bright yellow. With increasing GS content in the polymer, its color changed from light to intense yellow (Figure 7a). This corresponded to the increasing intensity of the GS peak in the UV–Vis spectra of the CS@GS and CS@GSA derivatives (Figure 7b,c). In general, the UV–Vis spectra of GS solutions show three main bands, whose position and intensity depend on the tautomer form of GS, solvent, and storage time [31]. For example, the spectrum of the freshly prepared ethanolic solution of GS at a concentration of 0.025 mg/mL showed two bands at 376 and 292 nm and one at <260 nm, among which the band at 376 nm was sensitive to GS concentration (Supplementary Materials; Figure S1). In the case of CS@GS and CS@GSA derivatives, the band at 376 nm attributed to the π → π* transition was observed at 426 nm. This shift towards longer wavelengths was likely caused by H-bonding interactions, as well as the specific configuration of the energy level in phenolic compound-conjugated polysaccharides [31,32]. Although the absorbance of bands at 426 and 323 nm increased linearly with increasing amount of GS added to the reaction mixture (Figure 7c,d), an increase of ~25% in peak absorbance in the CS@GSA spectrum indicated enhanced grafting efficiency when using GSA. The similar solubility of GS and GSA in ethanol/2% AA (confirmed by comparable absorbance; Figure S1c,d) suggested a supporting role of acetate salt in GS grafting, e.g., via intramolecular interactions.

**Figure 8:**
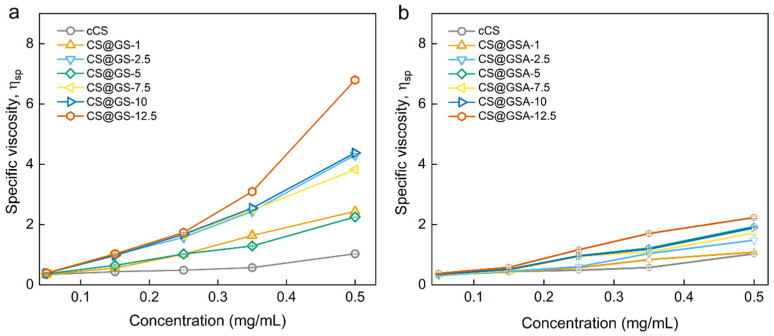
Dependence of specific viscosity on the concentration of (**a**) CS@GS and (**b**) CS@GSA derivatives in 2% AA.

Interestingly, the significant difference between CS@GS and CS@GSA series was observed in terms of polymer solubility and *η*_sp_ of polymer solutions. Regardless of using GS or GSA, the solubility of CS derivatives at RT was poorer compared to cCS, and deteriorated with increasing content of GS (Figure S2a,b). In addition, polymers containing GS were characterized by lower solubility in 2% AA than those prepared using GSA. This agreed with observation of *η*_sp_ (Figure 8). For CS@GS derivatives, the *η*_sp_ depended on the GS content and these differences increased with increasing concentration of the polymer solution (Figure 8a). For example, while at a concentration of 2.5 mg/mL, the *η*_sp_ of CS@GS-12.5 was less than two-fold higher than that observed for CS@GS-1, at a concentration of 0.5 mg/mL, this difference was almost seven times higher. In contrast, the difference between various CS@GSA derivatives was no higher than two-fold in the concentration range from 2.5 to 0.5 mg/mL (Figure 8b). Although the exact *M*_w_ of CS derivatives has not been determined, *η*_sp_ for CS@GS-12.5 was relatively close to the value observed for hvCS. This indicated a significant effect of GS on the *M*_w_ of CS@GS derivatives.

**Figure 9:**
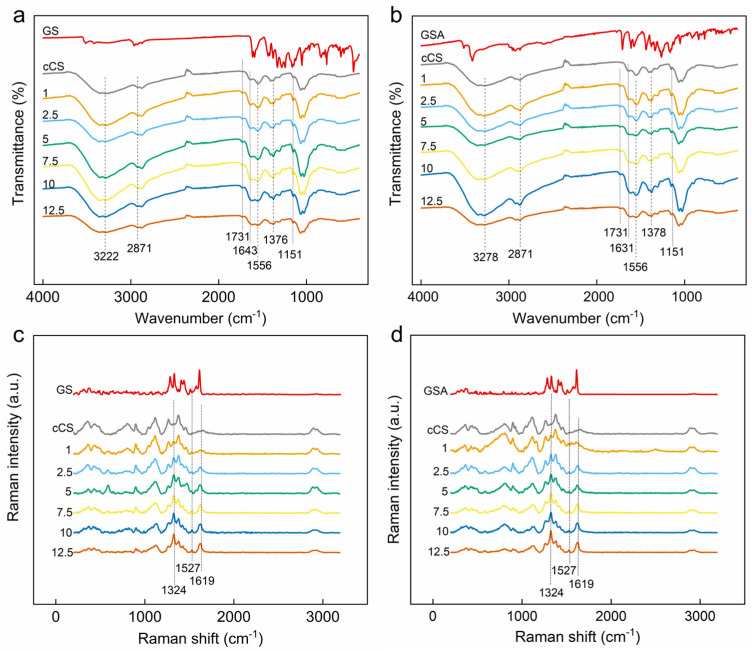
(**a**,**b**) ATR-FTIR and (**c**,**d**) Raman spectra of (**a**,**c**) CS@GS and (**b**,**d**) CS@GSA derivatives.

The ATR-FTIR spectra of CS@GS and CS@GSA derivatives were very consistent with the cCS spectrum in terms of peak positions and intensity (Figure 9a,b). No bands typical for GS were observed, likely due to their overlapping with much stronger CS bands. The lack of visible GS bands made it impossible to predict the type of bonding between GS and CS [33]. The only difference between the spectra of cCS and its derivatives was a band at 1731 cm^−1^ assigned to C=O. According to our previous observation, the position of this peak corresponded well to C=O in acetic acid [16]. Noteworthy, the peak at 1731 cm^−1^ was also present in the spectrum of GSA, but was not observed in the spectrum of GA, which also indicated acetic acid as its origin.

As a result of CS modification with GS, three new bands typical for GS appeared in the Raman spectra of CS@GS and CS@GSA derivatives; they intensified with increasing GS content (Figure 9c,d). A band at 1619 cm^−1^ was assigned to both vibrations of C=C in the phenolic rings and the deformation of the COH angle of the GS hydroxyls. Bands at 1524 and 1324 cm^−1^ were assigned to the ring stretching [34].

**Figure 10:**
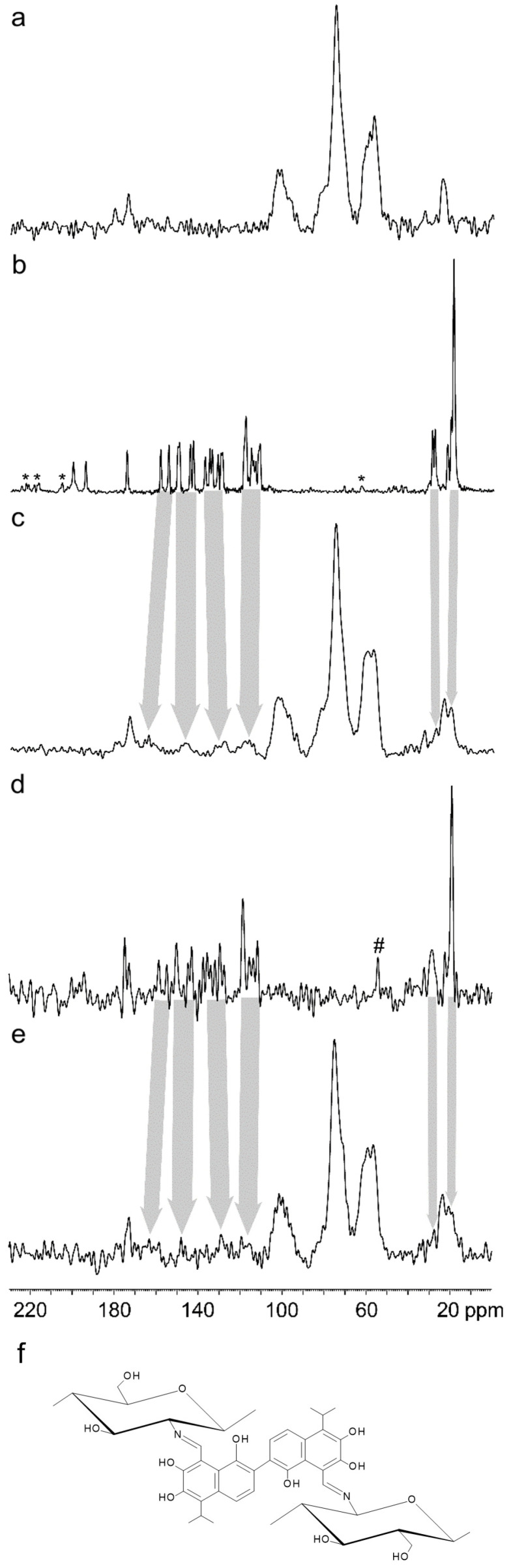
^13^C CP/MAS NMR spectra of (**a**) cCS, (**b**) GS, (**c**) CS@GS, (**d**) GSA, and (**e**) CS@GSA. (**f**) Schematic representation of CS crosslinking by GS via the C=N bond. Asterisks (*) denote spinning sideband signals and hash (#) corresponds to impurities.

In the ^13^C CP/MAS NMR spectra of CS@GS-12.5 and CS@GSA, new broad signals typical for GS appeared at 20, 27, 118, 130, 147, and 164 ppm, confirming the incorporation of GS into the CS backbone (Figure 10a–e). Similar spectra were observed for high- and low-molecular weight CS-Schiff base derivatives with GS [35]. The disappearance of the signals typical for the aldehyde carbons in CS (at 194.5 and 200.3 ppm) in combination with the appearance of a new signal at slightly lower frequency (~170 ppm) confirmed the bonding of GS via the C=N bond [36]. In addition, the broadening of GS-related signals suggested a high immobilization of GS molecules in the structure of the derivatives, consequently indicating crosslinking of the CS (Figure 10b–f).

According to the results of elemental analysis of the CS@GA series, the C content increased and the N content decreased with increasing amount of GS added to the reaction mixture; however, this correlation was only initially linear (Table 1). In the case of CS@GSA series, the contribution of C and N in polymers increased proportionally only up to the CS@GSA-5 polymer, after which a small drop in the C/N ratio was observed. Still, both the CS@GS-12.5 and CS@GSA-12.5 derivatives showed the highest and yet a similar C/N ratio (7.41 and 7.78, respectively). Importantly, the results of elemental analysis did not correspond with the results of UV–Vis and Raman spectroscopy, which showed peak intensity of GS-specific maxima increasing proportionally with the amount of GS added to the reaction mixture. This was likely caused by the presence of residual AA, which was not completely removed. However, the higher C content in the CS@GSA-12.5 agreed with the higher efficacy of GS grafting, as shown by the UV–Vis spectroscopy.

**Figure 11:**
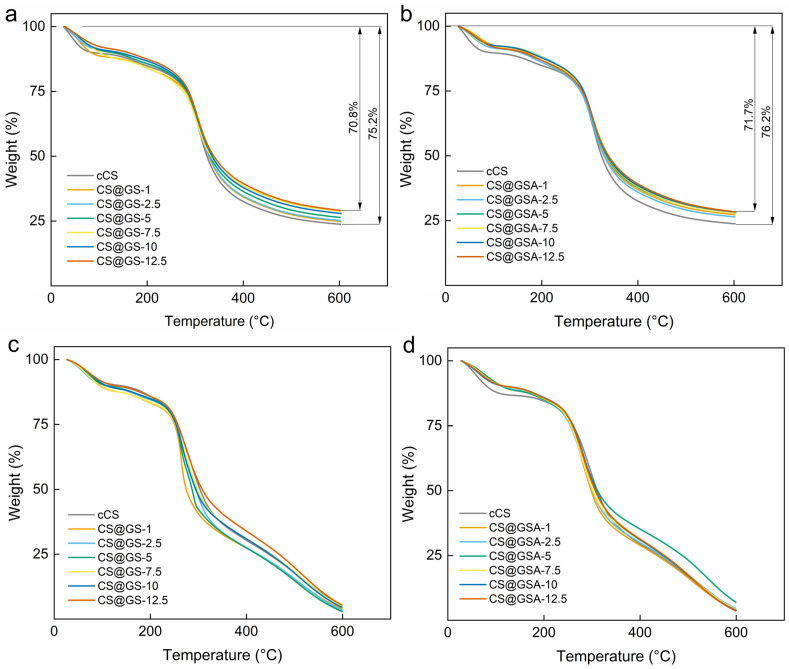
TGA curves of (**a**,**c**) CS@GS and (**b**,**d**) CS@GSA derivatives in (**a**,**b**) nitrogen and (**c**,**d**) air atmosphere.

The thermal decomposition of CS@GS and CS@GSA derivatives largely mimicked that of cCS (Figure 11). Interestingly, the observed difference between weight losses in nitrogen of the CS@GS series was noticeably smaller compared to those of the CS@GSA series. This agreed with the *η*_sp_ of the polymers, indicating that the degree of polymer crosslinking influenced the thermal stability. In addition, the thermal decomposition of most CS derivatives in air was shifted to lower temperatures, which excluded the protective role of GS against oxidative degradation of the polymer.

### 2.3. Antioxidant Properties of GS-Modified CS Derivatives

In the literature, there are only a few studies describing chitosan with bound GS or its derivatives that have significant antioxidant and/or cytotoxic properties. For example, CS@GS Schiff bases exhibited increased radical scavenging activity and altered thermal properties [36]. In this report, two methods were used to evaluate the antioxidant properties of the CS@GS and CS@GSA derivatives: Folin–Ciocalteu (F-C) method and 2,2-diphenyl-1-picrylhydrazyl (DPPH) assay. The F-C method enables one to determine the total phenolic content (expressed as gallic acid equivalent - *GAE*), while the DPPH assay evaluates the ability of the compounds to scavenge free radicals.

**Figure 12:**
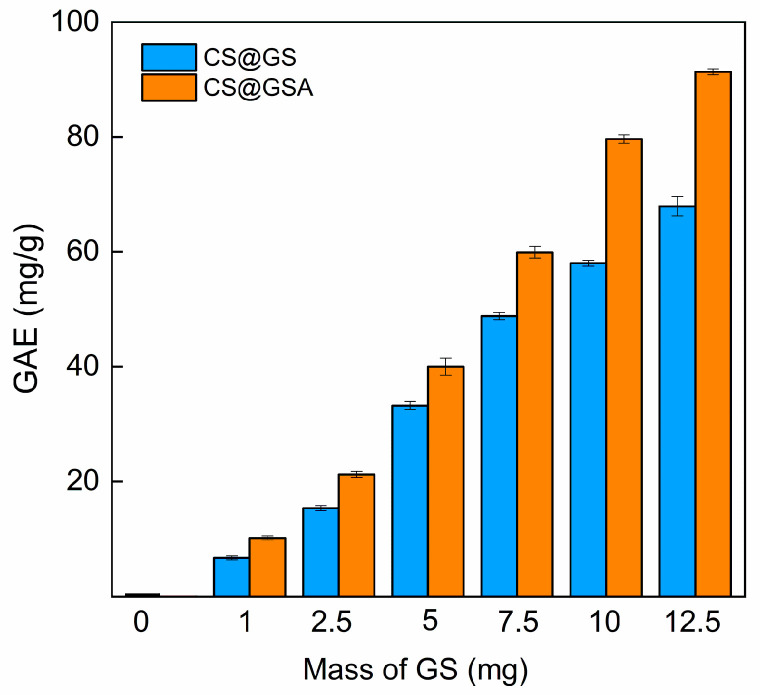
Dependence of the total phenolic content in CS@GS and CS@GSA derivatives on the amount of GS added to the reaction.

Importantly, the limitation of the F-C method is the lack of specificity. For example, sugars were mentioned as one of the factors interfering with the determination of total phenolic content, which explains the non-zero *GAE* value obtained for cCS [37]. In both series, the *GAE* values raised with increasing GS content (Figure 12); however, the CS@GSA derivatives were characterized by higher GAEs compared to those of their CS@GS counterparts. For instance, *GAE*s for CS@GS-12.5 and CS@GSA-12.5 were 68 and 91 mg/g of polymer, respectively. It is noteworthy that the higher the GS content, the greater the difference. This agreed with the UV–Vis observations, confirming the higher efficacy of GS grafting in this series.

**Figure 13:**
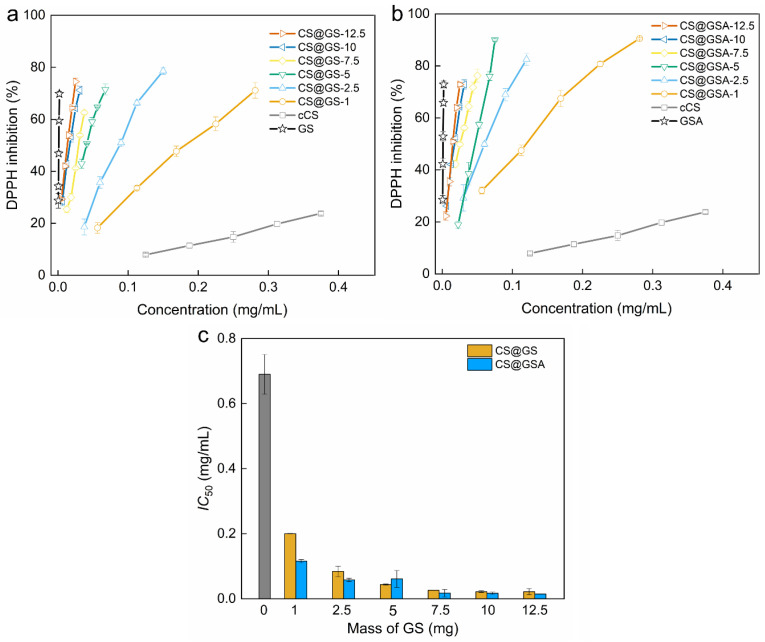
Dependence of DPPH inhibition on the concentration of (**a**) cCS and CS@GS and (**b**) cCS and CS@GSA derivatives. (**c**) Half-maximal inhibitory concentration (*IC*_50_) of cCS (grey bar), CS@GS, and CS@GSA derivatives.

Similarly, the ability to scavenge free radicals increased with increasing polymer concentration, as well as with the enhanced GS content (Figure 13). The derivatives with the lowest GS content, CS@GS-1 and CS@GSA-1, had half-maximal inhibitory concentration (*IC*_50_) of 0.20 and 0.12 mg/mL, respectively, which were significantly lower values compared to that of cCS, equaling to 0.7 mg/mL. In turn, polymers with the highest GS content, showed *IC*_50_ = 0.02 mg/mL for CS@GS-12.5 and CS@GSA-12.5 derivatives. All derivatives exhibited strong free-radical scavenging activity, slightly lower than for pure GS, yet well comparable to the antioxidant performance of other reported GS-containing CS [36,38].

## 3. Materials and Methods

### 3.1. Materials

Hydrogen peroxide (30%), ethanol (EtOH) for UV–Vis spectroscopy, and glacial acetic acid used to prepare 2% acetic acid (AA) were purchased from Lach-Ner (Neratovice, Czech Republic). 2,2-Diphenyl-1-picrylhydrazyl (DPPH), *L*-ascorbic acid (LAA), Folin–Ciocalteu (F-C) reagent, gallic acid, and highly viscous chitosan (hvCS) from crab shells were purchased from Sigma-Aldrich (St. Louis, MO, USA). (−)-Gossypol (GS) and (−)-gossypol acetate (GSA) were purchased from Gibco-Thermo Fischer Scientific (Waltham, MA, USA). All chemicals were used as received without further purification. The ultrapure water used for the dialysis of polymers was produced by the Milli-Q IQ 7000 system (Merck Millipore; Burlington, MA, USA).

### 3.2. Methods

#### 3.2.1. Synthesis of Chitosan Derivatives

CS was modified with GS by free-radical grafting using GS or GSA. Briefly, hvCS (100 mg) was dissolved in 2% AA (10 mL), homogenized at room temperature (RT) overnight, and mixed with EtOH (10 mL). GS and/or GSA was dissolved in EtOH (5 mL). Separately, LAA (11 mg; 0.0625 mmol) was dissolved in water (0.54 mL), mixed with 30% hydrogen peroxide (0.02 mL; 0.196 mmol), and after 5 min added to the CS solution. After 7 min, GS or GSA was added to the CS solution, and the mixture was stirred at RT for 24 h. To prepare a series of CS derivatives of increasing GS content, the amount of GS in the reaction mixture was increased accordingly (Table 2). Finally, the GS-grafted CSs were dialyzed (regenerated cellulose membrane; MWCO = 14,000 Da; Sigma-Aldrich) for 48 h against water, which was changed at least 8 times, and then lyophilized. To avoid GS photodecomposition and oxidation, the procedure was conducted in the dark under an argon atmosphere. The final CS-derivatives were stored at −20 °C and denoted as CS@GS-X and CS@GSA-X, respectively, where X corresponded to the mass of pure GS used (Table 2). The control CS (cCS) was prepared accordingly, but without GS.

#### 3.2.2. Physicochemical Characterization of the Polymers

The specific viscosity (*η*_sp_) was evaluated as a function of CS@GS or CS@GSA concentration. The measurements were performed using an Ubbelohde glass capillary viscometer with polymer solutions prepared in 2% AA at RT. The flow time of each solution and pure solvent was recorded and the specific viscosity *η*_sp_ was calculated using Equation (1):

*η*_sp_ = *t*_pol_/*t*_solv_ − 1 (1)

where *t*_pol_ and *t*_solv_ were the average flow times for the polymer solutions and the solvent, respectively. All measurements were performed in triplicate. To determine the critical solubility of hvCS, cCS, and GS-modified CS, polymers were dissolved at varying concentrations in 2% AA at RT. The critical solubility was defined as the highest concentration at which the polymer was completely dissolved.

ATR-FTIR spectra were collected with a Bruker Tensor 27 apparatus (Ettlingen, Germany) equipped with a mercury cadmium telluride detector and a Specac MKII Golden Gate Single Reflection ATR system (Orpington, UK) with a diamond crystal and an angle of incidence of 45°. The spectra were collected in the 400–4000 cm^−1^ range with a resolution of 4 cm^−1^ and 64 accumulations.

Raman spectra were measured using a Renishaw inVia Qontor Raman microspectrometer (Gloucestershire, UK) connected to a Leica DM2700 microscope (Wetzlar, Germany). The light source was a HPNIR laser with wavelength of 785 nm. A long-range objective focused the laser beam at 50× magnification. The diffraction grating had 1200 slits per 1 mm. The power was adjusted to prevent possible influence of the polymers. The exposure time was 10 s and the number of accumulations for a single spectrum was 4. Five spectra of hvCS, cCS, as well as CS@GS and CS@GSA derivatives, were collected in the 3200–200 cm^−1^ range and averaged. The WiRE v.5.5 Renishaw software was used to remove cosmic rays, perform baseline correction, and normalize spectra.

Solid-state NMR spectra were recorded using a Bruker AVANCE III HD spectrometer at 11.7 T (Ettlingen, Germany). The 4 mm cross-polarization magic angle spinning (CP/MAS) probe was used for ^13^C experiments at a Larmor frequency of 125.783 MHz. The ^13^C CP/MAS NMR spectra were acquired at a rotation frequency of 11 kHz using 2 ms CP contact time, 10 s recycle delay, and 10–20 k scans. High-power ^1^H decoupling (SPINAL64) was used for the removal of heteronuclear ^1^H and ^13^C coupling. Polymers were packed into ZrO_2_ rotors and subsequently kept at RT. All measurements were conducted at 298 K under active cooling to compensate for frictional heating caused by the spinning. The ^13^C NMR chemical shift was calibrated using α-glycine (carbonyl signal at 176.03 ppm) as an external standard [39]. The spectra were processed using Bruker TopSpin 4.1.1 software. Degree of deacetylation (*DDA*) for hvCS was calculated using Equation (2):

(2) $$DDA=100-(\frac{I\left({CH}_{3}\right)}{\frac{I\left(C_{1}\right)+I\left(C_{2}\right)+I\left(C_{3}\right)+I\left(C_{4}\right)+I\left(C_{5}\right)+I\left(C_{6}\right)}{6}})\times 100\;(\%)$$

where *I*(CH_3_) was the integral intensity of the resonance of methyl carbon and *I*(C_1_)–*I*(C_6_) were integral intensities of the resonances of the ring carbons [40].

C, H, and N analysis was performed by a Flash Smart™ analyzer from Thermo Fisher Scientific (Waltham, MA, USA). Results were expressed as the mean of two independent measurements. Additionally, cCS and GS-containing CS derivatives were neutralized before measurements to remove residual AA. The *DDA* for hvCS and cCS was calculated according to the literature [41] from Equation (3):

(3) $$DDA=\left[1-\frac{\left(\frac{C}{N}-5.145\right)}{6.81-5.145}\right]\times 100\;(\%)$$

where (*C*/*N*) was the ratio in the hvCS or cCS.

UV–Vis spectra of the polymers in 2% AA were collected by a Specord 210 spectrophotometer (Analytik Jena; Jena, Germany) in the range of 260–550 nm at a speed of 10 nm/s.

Thermal stability of the CSs was evaluated using a thermogravimetric analysis (TGA) performed on a Pyris 1 thermogravimetric analyzer (PerkinElmer; Waltham, MA, USA). The measurements were conducted in a temperature range of 30–600 °C with a heating rate of 10 °C/min and in air and nitrogen.

#### 3.2.3. Characterization of Antioxidant Properties

The antioxidant properties of cCS CS@GS and CS@GSA derivatives were investigated using two methods: Folin–Ciocalteu (F-C) test and DPPH assay.

In the F–C test, the total phenolic content was expressed as gallic acid equivalent (*GAE*). To determine *GAE*, polymers were dissolved in 2% AA (cCS, CS@GS/GSA-1-5 at 3 mg/mL, CS@GS/GSA-7.5 at 2.5 mg/mL, CS@GS/GSA-10 at 1.5 mg/mL, and CS@GS/GSA-12.5 at 1 mg/mL). The polymer solution (50 μL) was mixed with water (1.55 mL), supplemented with the F-C reagent (100 μL), and shaken for 140 s, which was followed by the addition of sodium carbonate solution (300 μL). After 1.5 h, the absorbance was measured at 765 nm using a Specord 250 spectrophotometer (Analytik Jena; Jena, Germany) against a blank solution. Each measurement was performed in triplicate. The final value was expressed as a mean of three independent measurements ± standard deviation (*SD*). The gallic acid calibration curve in the concentration range of 0–0.5 mg/mL (0.05, 0.1, 0.15, 0.25, and 0.5 mg/mL) and Equation (4) was used for *GAE* calculations:

(4) $$GAE(mg/g)=\frac{\frac{\left(A_{sample}-b\right)}{a}\cdot V_{extract}}{m_{polymer}}\times 1000$$

where *A* was the absorbance of the polymer test solution at 765 nm, *b* was the y-intercept of the calibration curve, *a* was the slope of the calibration curve, *V* was the volume of the polymer solution (mL), and *m*_polymer_ was the mass of the polymer (mg).

For the DPPH assay, a stock solution of DPPH (3.94 mg) in EtOH (100 mL) was left in the dark for 2 h and a series of cCS, CS@GS, and/or CS@GSA derivative solutions of different concentrations was prepared in 2% AA, i.e., cCS at 0.125–0.375 mg/mL, CS@GS/GSA-1 at 0.056–0.281 mg/mL, CS@GS/GSA-2.5 at 0.03–0.15 mg/mL, CS@GS/GSA-5 at 0.022–0.075 mg/mL, CS@GS/GSA-7.5 at 0.018–0.05, CS@GS/GSA-10 at 0.031–0.006 mg/mL, and CS@GS/GSA-12.5 at 0.025–0.005 mg/mL. The DPPH assay of pure GS in EtOH was performed in the concentration range of 0.0005–0.002 mg/mL; for GSA, the assay was performed identically with the correction of concentrations according to the presence of AA. For the test, EtOH (1.2 mL) was mixed with the tested solution (0.2 mL), vortexed for 10 s, and mixed with DPPH stock solution (0.6 mL). After 30 min, the absorbance was measured in the range of 400–700 nm (speed 5 nm/s) using a Specord 250 UV spectrometer. The peak maximum was read at 517 nm. Control was prepared by mixing DPPH stock solution (600 µL) and EtOH (1.4 mL). Each measurement was performed in triplicate. DPPH inhibition was determined from Equation (5):

(5) $${DPPH}_{inhibition}=\frac{A_{0}-A_{s}}{A_{0}}\times 100\;(\%)$$

where *A*_0_ and *A*_s_ were the absorbances of DPPH (control) and the tested solution, respectively. A half-maximal inhibitory concentration (*IC*_50_) was calculated from the linear dependence of DPPH inhibition on the sample concentration from Equation (6):

(6) $${IC}_{50}=(\frac{A_{0}}{2}-b)/a$$

where *A*_0_ was the absorbance of the DPPH (control) solution, *b* was the intercept, and *a* was the slope of the linear regression obtained. All values are expressed as a mean ± *SD* of three independent measurements.

## 4. Conclusions

Chitosan (CS) is a unique natural polycationic biopolymer valued for its biodegradability, biocompatibility, mucoadhesiveness, and low toxicity, with broad biomedical applications including for wound healing, tissue engineering, and targeted drug and gene delivery. Also gossypol (GS), a naturally occurring polyphenolic compound, exhibits notable antiinflammatory and antitumor activity, contraceptive properties, and metal chelating ability. In this report, we have advantageously combined the unique properties of both anticancer compounds to achieve a prospective synergistic effect against tumor cells. For this reason, we synthesized CS derivatives containing GS for the first time using free-radical grafting with two forms of GS: pure GS and its acetate salt GSA. This ensured good control over the amount of GS incorporated into the CS backbone. Treatment with LAA/H_2_O_2_ solution primarily affected the structural and thermal characteristics of polymers, while the form of grafting GS had a significant effect on crosslinking density. Importantly, grafting CS with GSA resulted in improved solubility of CS and significantly higher grafting efficiency, which was reflected in the enhanced antioxidant activity of the CS@GSA derivatives compared to CS@GS. Grafting of GS on CS thus critically increased the bioavailability of GS in aqueous media required for biomedical applications, primarily in cancer therapies. Accordingly, the proposed grafting approach represents a straightforward, environmentally friendly, and universal strategy for developing chitosan-based drug conjugates. In addition, the distinct mechanisms of action of GS and CS increase the chances of the developed system being effective against multidrug-resistant cancer; however, the toxicity of these new materials needs to be further considered.

## Figures and Tables

**Table 1:** Elemental composition of hvCS, cCS, and gossypol-modified CS derivatives.

Polymer	C (wt.%)	H (wt.%)	N (wt.%)	C/N
hvCS	40.78	6.99	7.42	5.49
cCS	37.17	6.47	6.67	5.57
CS@GS-1	37.33	6.38	6.50	5.74
CS@GS-2.5	36.11	6.02	5.74	6.29
CS@GS-5	35.61	6.40	5.64	6.32
CS@GS-7.5	37.85	6.39	5.86	6.46
CS@GS-10	35.22	6.15	4.84	7.28
CS@GS-12.5	31.16	6.53	4.21	7.41
CS@GSA-1	36.55	5.96	6.12	5.98
CS@GSA-2.5	35.95	6.18	5.70	6.31
CS@GSA-5	33.97	5.88	4.88	6.96
CS@GSA-7.5	34.55	6.39	5.07	6.82
CS@GSA-10	35.98	6.52	5.33	6.75
CS@GSA-12.5	31.69	6.35	4.08	7.78

**Table 2:** Amounts of hvCS, GS, and GSA used for the preparation of derivatives.

Polymer	hvCS (mg)	GS (mg)	GSA (mg) *
cCS	100	0	——————
CS@GS-1	100	1	——————
CS@GS-2.5	100	2.5	——————
CS@GS-5	100	5	——————
CS@GS-7.5	100	7.5	——————
CS@GS-10	100	10	——————
CS@GSA-12.5	100	12.5	——————
CS@GSA-1	100	——————	1.12
CS@GSA-2.5	100	——————	2.78
CS@GSA-5	100	——————	5.58
CS@GSA-7.5	100	——————	8.37
CS@GSA-10	100	——————	11.16
CS@GSA-12.5	100	——————	13.95

* Based on GS/AA ratio.

## Data Availability

The original contributions presented in this study are included in the article and Supplementary Materials. Further inquiries can be directed to the corresponding authors.

## Supplementary Materials

The following supporting information can be downloaded at: https://www.mdpi.com/article/10.3390/ijms262311721/s1.

## Abbreviations

The following abbreviations are used in this manuscript:

ATR-FTIR	Attenuated total reflectance Fourier-transform infrared spectroscopy
AA	Acetic acid
CS	Chitosan
CS@GS(A)	Gossypol-modified chitosan prepared using GS or GSA
cCS	Control chitosan
*DDA*	Degree of deacetylation
DPPH	2,2-Diphenyl-1-picrylhydrazyl
EtOH	Ethanol
F-C	Folin–Ciocalteu
hvCS	Highly viscous chitosan
*IC*_50_	Half-maximal inhibitory concentration
*GAE*	Gallic acid equivalent
GS	Gossypol
GSA	Gossypol acetate
LAA	*L*-ascorbic acid
NMR	Nuclear magnetic resonance
RT	Room temperature
*SD*	Standard deviation
TGA	Thermogravimetric analysis
*η*_sp_	Specific viscosity

## References

[1] De Oliveira I., Santos-Buelga C., Aquino Y., Barros L., Heleno S.A. (2025). New frontiers in the exploration of phenolic compounds and other bioactives as natural preservatives. Food Biosci..

[2] Paunovic D., Rajkovic J., Novakovic R., Grujic-Milanovic J., Mekky R.H., Popa D., Calina D., Sharifi-Rad J. (2023). The potential roles of gossypol as anticancer agent: Advances and future directions. Chin. Med..

[3] Liu Y., Wang L., Zhao L., Zhang Y. (2022). Structure, properties of gossypol and its derivatives—From physiological activities to drug discovery and drug design. Nat. Prod. Rep..

[4] Bera R., Bandyopadhyay R., Debnath B., Dutta G., Sugumaran A. (2025). Review on various activator-assisted polymer grafting techniques for smart drug delivery applications. RSC Adv..

[5] Mohammed A.S.A., Naveed M., Jost N. (2021). Polysaccharides; Classification, chemical properties, and future perspective applications in fields of pharmacology and biological medicine (A review of current applications and upcoming potentialities). J. Polym. Environ..

[6] Kołodziejska M., Jankowska K., Klak M., Wszoła M. (2021). Chitosan as an underrated polymer in modern tissue engineering. Nanomaterials.

[7] Li X., Dong W., Nalin A.P., Wang Y., Pan P., Xu B., Zhang Y., Tun S., Zhang J., Wang L.-S. (2018). The natural product chitosan enhances the anti-tumor activity of natural killer cells by activating dendritic cells. Oncoimmunology.

[8] Wimardhani Y.S., Suniarti D.F., Freisleben H.J., Wanandi S.I., Siregar N.C., Ikeda M.A. (2014). Chitosan exerts anticancer activity through induction of apoptosis and cell cycle arrest in oral cancer cells. J. Oral Sci..

[9] Jiang Y., Yu X., Su C., Zhao L., Shi Y. (2019). Chitosan nanoparticles induced the antitumor effect in hepatocellular carcinoma cells by regulating ROS-mediated mitochondrial damage and endoplasmic reticulum stress. Artif. Cells Nanomed. Biotechnol..

[10] Hussain Shah S.N., Zulcaif Syed A., Syed A., Aslam A., Zafar N., Arif A. (2024). Development of film forming gel for the delivery of 5-flurouracil: In-vitro/ex-vivo evaluation. Polym. Bull..

[11] Chen S., Deng J., Zhang L.-M. (2021). Cationic nanoparticles self-assembled from amphiphilic chitosan derivatives containing poly(amidoamine) dendrons and deoxycholic acid as a vector for co-delivery of doxorubicin and gene. Carbohydr. Polym..

[12] Pan Z., Gao Y., Heng L., Liu Y., Yao G., Wang Y., Liu Y. (2013). Amphiphilic *N*-(2,3-dihydroxypropyl)–chitosan–cholic acid micelles for paclitaxel delivery. Carbohydr. Polym..

[13] Sood A., Gupta A., Bharadwaj R., Ranganath P., Silverman N., Agrawal G. (2022). Biodegradable disulfide crosslinked chitosan/stearic acid nanoparticles for dual drug delivery for colorectal cancer. Carbohydr. Polym..

[14] Wan Yusof W.R., Awang N.Y.F., Azhar Laile M.A., Azizi J., Awang Husaini A.A.S., Seeni A., Wilson L.D., Sabar S. (2023). Chemically modified water-soluble chitosan derivatives: Modification strategies, biological activities, and applications. Polym.-Plast. Technol. Mater..

[15] Oliver S., Vittorio O., Cirillo G., Boyer C. (2016). Enhancing the therapeutic effects of polyphenols with macromolecules. Polym. Chem..

[16] Świętek M., Lu Y.-C., Konefał R., Ferreira L.P., Cruz M.M., Ma Y.-H., Horák D. (2019). Scavenging of reactive oxygen species by phenolic compound-modified maghemite nanoparticles. Beilstein J. Nanotechnol..

[17] Liu J., Wen X.-Y., Lu J.-F., Kan J., Jin C.-H. (2014). Free radical mediated grafting of chitosan with caffeic and ferulic acids: Structures and antioxidant activity. Int. J. Biol. Macromol..

[18] Diao Y., Yu X., Zhang C., Jing Y. (2020). Quercetin-grafted chitosan prepared by free radical grafting: Characterization and evaluation of antioxidant and antibacterial properties. J. Food Sci. Technol..

[19] Wu T., Wu C., Xiang Y., Huang J., Luan L., Chena S., Hu Y. (2016). Kinetics and mechanism of degradation of chitosan by combining sonolysis with H2O2/ascorbic acid. RSC Adv..

[20] Liu J., Pu H., Zhang X., Xiao L., Kan J., Jin C. (2018). Effects of ascorbate and hydroxyl radical degradations on the structural, physicochemical, antioxidant and film forming properties of chitosan. Int. J. Biol. Macromol..

[21] Mittal A., Singh A., Hong H., Benjakul S. (2023). Chitooligosaccharides from shrimp shell chitosan prepared using H_2_O_2_ or ascorbic acid/H_2_O_2_ redox pair hydrolysis: Characteristics, antioxidant and antimicrobial activities. Int. J. Food Sci. Technol..

[22] Tapia A., Seña R., Zambrano H., Paredes V. (2025). Extraction and characterization of chitosan obtained from shells of crab (Callinectes bocourti and Callinectes sapidus). Int. J. Biol. Macromol..

[23] Hong S., Choi H., Jo S., Kim M.-J., Lee S., Ahn S., Lee J. (2019). Modification of chitosan using hydrogen peroxide and ascorbic acid and its physicochemical properties including water solubility, oil entrapment and in vitro lipase activity. Int. J. Food Sci. Technol..

[24] Zając A., Hanuza J., Wandas M., Dymińska L. (2015). Determination of *N*-acetylation degree in chitosan using Raman spectroscopy. Spectrochim. Act. Part A Mol. Biomol. Spectrosc..

[25] Zhang J., Xia W., Liu P., Cheng Q., Tahirou T., Gu W., Li B. (2010). Chitosan modification and pharmaceutical/biomedical applications. Mar. Drugs.

[26] Farion I.A., Burdukovskii V.F., Kholkhoev B.C., Timashev P.S., Chailakhyan R.K. (2018). Functionalization of chitosan with carboxylic acids and derivatives of them: Synthesis issues and prospects of practical use: A review. Express Polym. Lett..

[27] Ziegler-Borowska M., Chełminiak D., Kaczmarek H. (2015). Thermal stability of magnetic nanoparticles coated by blends of modified chitosan and poly(quaternary ammonium) salt. J. Therm. Anal. Calorim..

[28] Beda A., Yamada H., Egunov A., Ghimbeu C.M., Malval J.-P., Saito Y., Luchnikov V. (2019). Carbon microtubes derived from self-rolled chitosan acetate films and graphitized by joule heating. J. Mater. Sci..

[29] Moreno-Vásquez M.J., Valenzuela-Buitimea E.L., Plascencia-Jatomea M., Encinas-Encinas J.C., Rodríguez-Félix F., Sánchez-Valdes S., Carina Rosas-Burgos E., Ocaño-Higuera V.M., Graciano-Verdugo A.Z. (2017). Functionalization of chitosan by a free radical reaction: Characterization, antioxidant and antibacterial potential. Carbohyd. Polym..

[30] Qin C.Q., Du Y.M., Xiao L. (2002). Effect of hydrogen peroxide treatment on the molecular weight and structure of chitosan. Polym. Degrad. Stab..

[31] Wang L., Liu Y., Zhang Y., Yasin A., Zhang L. (2019). Investigating stability and tautomerization of gossypol—A spectroscopy study. Molecules.

[32] Zhang W., Sun J., Li Q., Liu C., Niu F., Yue R., Zhang Y., Zhu H., Ma C., Deng S. (2023). Free radical-mediated grafting of natural polysaccharides such as chitosan, starch, inulin, and pectin with some polyphenols: Synthesis, structural characterization, bioactivities, and applications—A review. Foods.

[33] Przybylski P., Bejcar G., Huczyński A., Schroeder G., Brzezinski B., Franz B. (2006). ^1^H- and ^13^C-NMR, FTIR, UV-VIS, ESI-MS, and PM5 studies as well as emission properties of a new Schiff base of gossypol with 5-methoxytryptamine and a new hydrazone of gossypol with dansylhydrazine. Biopolymers.

[34] Sánchez-Cortés S., García-Ramos J.V. (2000). Adsorption and chemical modification of phenols on a silver surface. J. Colloid. Interface Sci..

[35] Demetgül C., Beyazit N. (2018). Synthesis, characterization and antioxidant activity of chitosan-chromone derivatives. Carbohyd. Polym..

[36] Beyazit N., Çakran H.S., Cabir A., Akışcan Y., Demetgül C. (2020). Synthesis, characterization and antioxidant activity of chitosan Schiff base derivatives bearing (−)-gossypol. Carbohyd. Polym..

[37] Lawag I.L., Nolden E.S., Schaper A.A.M., Lim L.Y., Locher C. (2023). A modified Folin-Ciocalteu assay for the determination of total phenolics content in honey. Appl. Sci..

[38] Mohammed M.A., Amer N.M., Abdallah H.M.I., Saleh M.S. (2024). A comprehensive tool in recycling plant-waste of *Gossypium barbadense* L agricultural and industrial waste extracts containing gossypin and gossypol: Hepatoprotective, anti-inflammatory and antioxidant effects. Plant Methods.

[39] Morcombe C.R., Zilm K.W. (2003). Chemical shift referencing in MAS solid state NMR. J. Magn. Reson..

[40] Harish Prashanth K.V., Kittur F.S., Tharanathan R.N. (2002). Solid state structure of chitosan prepared under different *N*-deacetylating conditions. Carbohyd. Polym..

[41] Gupta K.C., Jabrail F.B. (2006). Effects of degree of deacetylation and cross-linking on physical characteristics, swelling and release behavior of chitosan microspheres. Carbohyd. Polym..

