# Supplementary material for: Modification of Chitosan with (−)-Gossypol and (−)-Gossypol Acetic Acid Using Free-Radical Grafting Method
Source: Int J Mol Sci. 2025 Dec 3;26(23):11721. doi: 10.3390/ijms262311721 (PMC12692262; doi:10.3390/ijms262311721)
Supplement: Supplementary file 1 [file ijms-26-11721-s001.zip › ijms-3980360-supplementary.pdf]

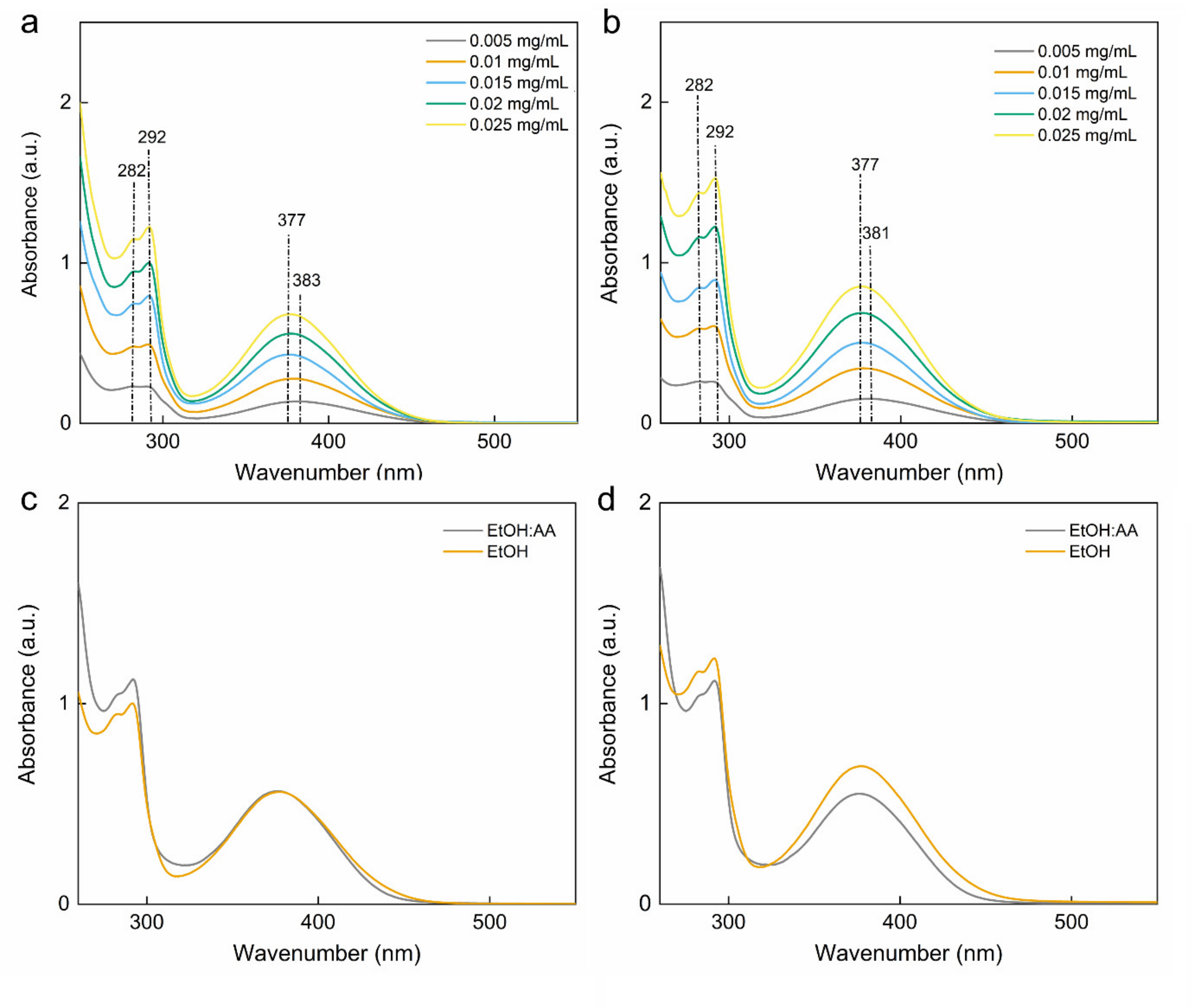

Figure S1. UV-Vis spectra of ethanolic solutions of (a, c) GS and (b, d) GSA at various concentrations.

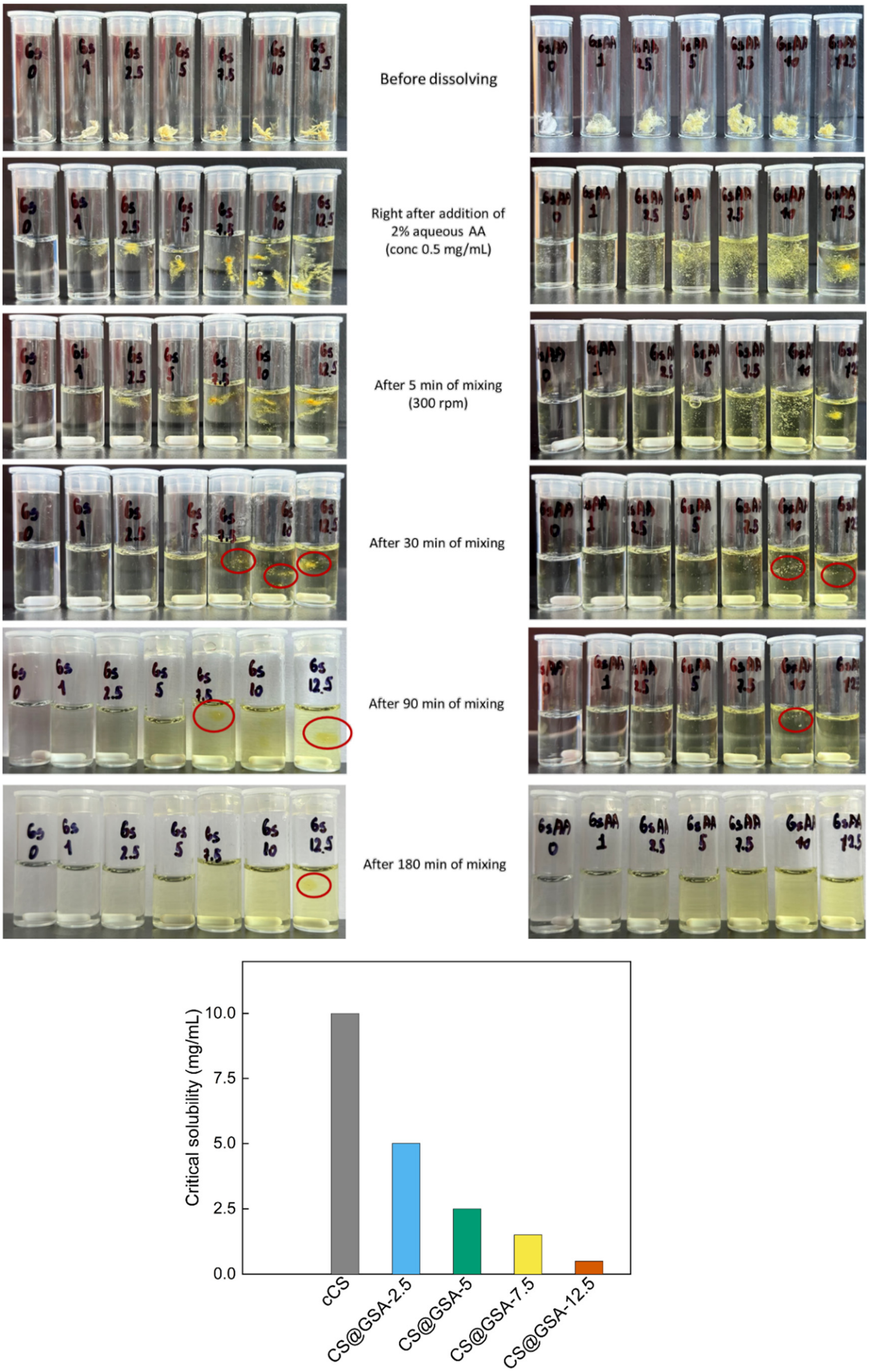

Figure S2. Solubility of cCS and CS@GS (left) and CS@GSA (right) in 2 % AA (0.05 mg/mL) at RT. Critical solubility of cCS and CS@GSA derivatives (bottom).
